# Bolder together: conformity drives behavioral plasticity in eastern gartersnakes

**DOI:** 10.1007/s10071-024-01859-5

**Published:** 2024-02-22

**Authors:** Morgan Skinner, Gokulan Nagabaskaran, Tom Gantert, Noam Miller

**Affiliations:** 1 Department of Psychology, Wilfrid Laurier University, 75 University Ave. West, Waterloo, ONT N2L 3C5 Canada; 2 School of Nursing, Fanshawe College, London, Ontario Canada

**Keywords:** Gartersnake, Personality, Conformity, Plasticity, Boldness, Sociality

## Abstract

Personality traits drive individual differences in behaviour that are consistent across time and context. Personality limits behavioural plasticity, which could lead to maladaptive choices if animals cannot adapt their behavior to changing conditions. Here, we assessed consistency and flexibility in one personality trait, boldness, across non-social and social contexts in eastern gartersnakes (*Thamnophis sirtalis sirtalis*). Snakes explored a novel open arena either alone or in a pair. Pairs were assigned based on the data from the solo trials, such that each snake was paired once with a bolder and once with a less bold partner. We predicted that snakes would conform when in a social context, displaying plasticity in their personality, and causing boldness scores to converge. We found that snakes were consistent within contexts (solo or paired), but changed their behavior across contexts (from solo to paired). Plasticity in boldness resulted from an interaction between conformity and repeatable individual differences in plasticity. In line with some data on other species, snakes conformed more when they were the less bold partner. Personality reflects a consistent bias in decision-making, but our results highlight that the cognitive processes that drive the expression of personality traits in behavior are flexible and sensitive to social context. We show that both consistency and plasticity combine to shape snake social behavior in ways that are responsive to competition. This pattern of behavior may be particularly beneficial for species in which group-living is seasonal.

**Supplementary Information:**

The online version contains supplementary material available at 10.1007/s10071-024-01859-5.

## Introduction

A behavior is considered to potentially be driven by a personality trait when it is exhibited consistently across time and contexts—when it is repeatable—and when it varies across individuals within a species (Dall et al. 2004; Sih et al. 2004; Wolf and Weissing 2012). By these criteria, numerous behaviors have been found to be repeatable, across a wide range of taxa (Bell et al. 2009), exposing traits such as boldness, exploration, activity, sociability, and aggression (Conrad et al. 2011; Wolf and Weissing 2012; Cabrera et al. 2021; Gartland et al. 2021). Though many studies have demonstrated consistency in the behaviors shaped by these traits across both time and context, researchers are increasingly showing that personality traits can also be flexible, under the right conditions (e.g., Jolles et al. 2019).

The extent to which behavior is plastic despite the apparent constraints imposed by personality is an active area of research (Brand et al. 2022; Cabrera et al. 2021; Meuthen et al. 2019; Thompson et al. 2018). There are obvious advantages to being able to adapt behavior to the current context, which is limited by the biases in action selection introduced by personality traits (Sih et al. 2004). Behavioral plasticity can be inferred from variability in individual behavior that reduces or eliminates the repeatability of personality traits (Brand et al. 2022), changes in the correlations between behaviors (Bell and Stamps 2004), or changes in the mean levels of a behavior across subgroups (Guayasamin et al. 2017; Skinner et al. 2022). For example, research on three-spined sticklebacks (*Gasterosteus aculeatus*) has demonstrated that personality traits vary across environments with different predation pressures (Bell 2005) and that plasticity in aggression can be induced by exposure to predation threat (Bell and Sih 2007). In addition, research suggests that individuals display consistent individual differences in behavioral flexibility (Dingemanse et al. 2010), such that flexibility itself may be considered a personality trait (Jolles et al. 2019). For example, female Ural Owls (*Strix uralensis*) display individual differences in plasticity of aggression related to changes in the abundance of prey (Kontiainen et al. 2009), and more exploratory great tits (*Parus major*) show more plasticity than less exploratory great tits with repeated exposure to a testing environment (Dingemanse et al. 2012). Importantly, exploring plasticity in personality traits may improve our understanding of the cognitive mechanisms that underlie personality (Stamps and Biro 2016; Boogert et al. 2018). Identifying the ecological, social, or developmental factors that modulate the expression of personality traits, and how they do so, will point towards the ways in which the behavioral biases that make up personalities affect behavior, under what circumstances they have an effect, and how flexible their influence is.

There is growing evidence to suggest that changes in social context may be particularly effective in inducing behavioral plasticity, suggesting that at least some cognitive processes affected by personality traits are also sensitive to social context (Castenheira et al. 2016; Montiglio et al. 2017; King et al. 2015). For example, Guayasamin et al. (2017) measured exploration of a novel tank in zebrafish (*Danio rerio*) paired with both more exploratory and less exploratory partners. They found that exploration increased when individuals were the less exploratory partner and that this plasticity was in part driven by individual differences in exploration flexibility. In other words, less exploratory fish altered their behavior to more closely match that of a partner, demonstrating plasticity in aid of conformity (Ioannou and Laskowski 2023). Plasticity across social contexts has also been demonstrated in lizards. For example, delicate skinks (*Lampropholis delicata*) show repeatability in boldness within non-social and social contexts, but not when boldness is compared across these two contexts (Brand et al. 2022). In addition, tree skinks (*Egernia striolata*) that were reared in isolation demonstrated plasticity in their sociability across social contexts (Riley et al. 2018).

Plasticity in personality traits caused by interactions with conspecifics could be driven by a range of social cognitive processes, such as social facilitation or imitation (Zentall 2006). If these processes promote more similar behaviors across group members than might be expected based on environmental conditions, the resulting collective behavior is often labelled conformity (Pike and Laland 2010; Webster and Ward 2010). Conformity has been studied predominantly in species that form tight social groups such as primates and fish (e.g., Humans, *Homo sapiens*, Morgan & Laland 2012; chimpanzees, *Pan troglodytes*, Whiten et al. 2005; zebrafish, Ayoub et al. 2019). Perhaps due to the perceived relationship between conformity and group cohesion (Lott and Lott 1961; Fonseca et al. 2018), it is often discussed in conjunction with complex group-living processes such as cultural transmission (Morgan and Laland 2012) and cooperation (Yang and Lan 2017). However, it has been suggested that the mechanisms of conformity need not be particularly complex (Webster and Ward 2010), and research has found rudimentary conformity in a wider variety of taxa than might be expected. For example, fruit flies (*Drosophila melanogaster*) conform to the oviposition sites of other individuals (Battesti et al. 2012) and shore crabs (*Carcinus maenas*) conform their activity level to match a less active conspecific (Fürtbauer and Fry 2018). These data raise an often overlooked aspect of conformity—that its particular expression may differ based on the social circumstances or ecology of the animal. For example, in a group of two individuals, conformity may be unidirectional, with one individual changing to match the behavior of another (e.g., Fürtbauer and Fry 2018), or there can be co-conformity, with both individuals adjusting to meet in the middle (e.g., King et al. 2015). Although certain patterns of conformity may predict behavior in group living animals such as primates and humans, animals with highly competitive and less cohesive social groups, such as snakes, may demonstrate alternate patterns of conformity. Research on these taxa can provide valuable insights into the types of social systems in which social cognitive processes can override typical behavioral patterns.

Gartersnakes are a medium-sized colubrid snake native to much of North America (Rossman et al. 1996). The eastern gartersnakes used in the current study were from Ontario, Canada. As such, these snakes would hibernate in groups during the colder months and emerge from hibernation to mate in groups during the spring (Rossman et al. 1996). After hibernation and mating, gartersnakes disperse for the remainder of their active season (Gregory and Steward 1975; Rossman et al. 1996). Gartersnakes are interesting model organisms for research on the effects of social context on behavior, as they do not form long-term social groups and seasonally transition between social contexts, alternating between aggregation and solitary dispersal. In addition, the gartersnake social system is highly competitive: mating occurs in large groups with multiple males often competing for opportunities to mate with a single female (Whittier et al. 1985; Shine et al. 2004), and snakes cannot share food, making conspecifics potential competitors for food (Yeager and Burghardt 1991). Furthermore, recent studies have shown that gartersnakes demonstrate consistency in their social interactions, with some snakes consistently more social than others, which suggests that they may develop association patterns to mitigate the effects of competition (Skinner and Miller 2020, 2022). Thus, gartersnake social cognition may be sensitive to social context, despite their seasonally facultative social system. Evidence of plasticity in personality in response to social interaction in gartersnakes would, therefore, suggest that conformity is more widespread across social systems than is currently assumed.

Here, we investigated to what extent gartersnakes are consistent in their boldness across both a non-social and two similar social contexts, in which they were either bolder or less bold than a conspecific in the same arena. We adopted a similar process to that utilized by Guayasamin et al. (2017) in zebrafish. We used data from non-social boldness assays to create pairs of snakes that varied in the direction and magnitude of their differences in boldness. We then ran these pairs of snakes through the same boldness assay. Each snake performed the paired assay twice; once with a bolder partner and once with a less bold partner (in a counterbalanced order). This procedure allowed for investigating relative boldness in a manner that is likely more common in a natural setting, as individuals likely encounter conspecifics that are both bolder and less bold than themselves. We hypothesized that snakes would either show repeatability in boldness across social contexts or display social conformity, adjusting their behavior to match that of their partner. We made no prediction as to whether the bolder or less bold partner would adjust their behavior to meet that of the other snake; both patterns have previously been observed in other taxa (Fürtbauer and Fry 2018; Munson et al. 2021).

## Methods

### Subjects and housing

Sixty-two eastern gartersnakes (35 M: 27 F) served as subjects in this experiment. The snakes were purchased from local breeders or donated by reptile zoos. They were acquired and tested in two separate batches. Snakes were neonates when acquired and were tested at ~ 10 months of age. The relatedness of the snakes was unknown. Subjects were housed in same-sex groups of 2–6, in 20 gallon glass aquariums (51 × 26 × 30.5 cm) with mesh lids. When they grew larger, they were transitioned to smaller groups of 2–3 housed in plastic boxes with mesh lids (46 × 31 × 17 cm). The housing room was temperature controlled (22 °C) with a 12 h light cycle (lights on from 7 am to 7 pm). Paper towel substrate was used, and clean water was provided daily. Subjects were fed chopped nightcrawlers (*Lumbricus* *terrestris*; Pagonis Live Bait, Toronto) with vitamin supplements (Zilla). Subjects had access to belly heat (30 °C) provided by heat tape (THGHeat) and shelters (14 cm × 10.2 cm × 5 cm high; Cornel’s World) on both the cool and warm sides of the tank.

### Apparatus

Both solo and paired boldness trials were held in the same arena, a Styrofoam box measuring 40.6 cm × 45.7 cm × 33 cm high (see Figure S1 and Video S1). For the solo trials, one black plastic reptile shelter, identical to the shelters in the home tank, was placed against the center of one long wall of the arena (Figure S1A). For the paired trials, two of the shelters were placed adjacent to each other, in the centre of one long wall of the arena (Figure S1B). Two shelters were used for the paired trials to reduce the possibility that snakes left shelter due to social avoidance. For both types of trials, we placed the testing arenas beside each other, so that four subjects could be run at a time. In this way, the number of snakes in the testing room was consistent across both paired and solo trials. It is unlikely that snakes could perceive any relevant information about snakes in the other arenas in the testing room (such as whether they were in or out of a shelter), but this made the testing room smell of snakes, like the housing room, which may have reduced stress overall. Arenas were covered with a clear sheet of acrylic to prevent escapes. All trials were recorded using a camcorder (Panasonic HC-V700) mounted above the arena. Sample trial videos are given in the SI (Videos S1 and S2).

### Procedure

The procedure was based on methods used by Guayasamin et al. (2017) to test for cross-context boldness plasticity in zebrafish, which we adapted using boldness assays that we have previously validated in gartersnakes (Skinner and Miller 2020; Skinner et al. 2022). Subjects received at least two solo boldness trials and two boldness trials with a partner. Scores from the final boldness trial were used to assign partners. Paired trials were performed with two different partners—one partner that was more bold and one that was less bold, with the order of these pairings counterbalanced across subjects. The testing procedure for the solo boldness trials is further described in Skinner and Miller (2022). Briefly, subjects were individually marked with 1–2 colored dots on their head using non-toxic nail polish (Adrianne K) prior to each experiment. Subjects’ identities were tracked using head and neck color patterns that differentiated them from their cage mates. Subjects were not tested on days when they were fed (Mondays and Thursdays) and completed no more than one assay per week, to limit arena habituation (Skinner and Miller 2020). Solo trial data for the first batch of subjects (*n* = 36) were collated from previously published data in which snakes were individually tested for boldness consistency across development (Skinner and Miller 2022). The remaining subjects (*n* = 26) were tested individually for boldness twice. Starting 2 weeks after completion of their final solo assay, each subject received two paired trials with different partners. The entire testing process occurred over approximately 4 weeks.

We paired subjects in the same manner as Guaysamin et al. (2017) paired fish, based on recent solo boldness scores. The direction of the differences in boldness varied, such that snakes were bolder than one partner (MB condition) and less bold than their other partner (LB condition). The magnitude of the boldness differences between the partners was also varied. Adopting the terminology of Guayasamin et al. (2017), we refer to this as the intra-pair boldness difference (IPBD). As some snakes were inevitably more bold or less bold than all other snakes, it was necessary to pair the three boldest and three least bold snakes in each testing group with only less bold and more bold partners, respectively. We used the data from these snakes, who only experienced either the MB or LB conditions, as a control for the remaining pair trials. We avoided pairing snakes with familiar conspecifics (i.e., cage mates).

In preparation for each assay, subjects were gently removed from their home tanks and placed in groups of two in a bucket with a clean paper towel in it (even for solo assays). Buckets were covered with clear plastic lids to prevent escapes. Subjects were transported in the buckets to the testing room, and spent no more than five minutes in the buckets before being placed into the arenas.

To begin the solo assays, subjects were placed close to the entrance of the shelter and allowed to slither into the shelter to start the trial (Skinner and Miller 2020; Skinner et al. 2022). For paired assays, the same process was followed but each subject was placed into their own shelter. Time spent outside of the shelter as a proportion of the session duration was used as a measure of boldness (Koenig and Ousterhout 2018; Jolles et al. 2016). Both solo and paired assays lasted 20 min, as previous research has demonstrated that this time frame is sufficient for assaying boldness in gartersnakes (Skinner and Miller 2020; Skinner et al. 2022). The arenas were thoroughly cleaned and dried between trials using 70% isopropyl alcohol (which was allowed to evaporate before the next trial began), water, and paper towels.

### Analysis

A custom ethologger was used to manually code all videos. The subject’s location was determined by clicking on the area of the arena it occupied, as determined by the position of its head. Subjects were classified as being either inside the shelter or outside it (when more than half of their body was visible outside the shelter). For the paired assays, time spent in either of the two shelters was collapsed into a single measure, for comparison with the solo assays. We additionally measured the latency until each snake’s first emergence from the shelter (in both solo and paired trials), and the number of visits they made to the shelter (as in Guayasamin et al. 2017). The videos were coded by authors MS and GN. Inter-rater reliability between the two coders was high with an intra class correlation of 0.97.Statistical analyses were performed in R (v4.02; R Core Team 2022). All proportion data were arcsine transformed. Other skewed variables were transformed using the *Guassianize* function from the *LambertW* package (Goerg 2023). Subjects were scored on the proportion of the trial they spent outside the shelter. Changes in boldness score (Δbold) were calculated as the difference between the solo trial score and each paired condition (MB and LB) divided by the maximum possible change (to ensure that the measure was independent of the initial score). To model changes across conditions (from solo to paired), we fit mixed-effect linear models using the *lme4* package (Bates et al. 2015). As we had multiple measurements for the same individual, all the models contained random intercepts for ‘individual’ nesting within ‘batch’. As prior research has shown that sex and weight can play important roles in gartersnake behavior (Skinner et al. 2022), we included Sex, Weight, and the interaction between the two in our model. To test for testing order effects, we used two models: first, we modeled the combined effects of trial number, pairing types (i.e., one MB and one LB trial; both trials LB; or both trials MB), and the interaction between the two on boldness plasticity; for the second model, we replaced ‘pairing types’ with ‘first partner types’ to see if being the more bold or less bold partner on the first pair trial changed absolute boldness plasticity across trials. To test overall effects, we used the ANOVA function on each model.

To estimate individuals’ repeatability across conditions, we used the *rptR* function with 1000 bootstraps per model. We tested for boldness repeatability across the two solo trials, between the two paired conditions (within context; LB–MB), and between the solo and each paired condition (across social contexts; Solo-LB and Solo-MB). In addition, we tested for repeatability in boldness plasticity—the change in boldness (Δbold)—across the two conditions (Δbold Solo-LB and Δbold Solo-MB). We included batch as a fixed effect in these models and, therefore, report R adjusted for batch (Radj|batch). In order for one model to converge, it had to be adjusted for both condition and batch (Radj|cond + batch). Along with repeatability, we report the between-individual and within-individual variance (Nakagawa and Schielzeth 2010).

## Results

### Repeatability

Boldness was significantly repeatable across solo trials (Radj|batch = 0.32, 95% CI [0.07, 0.52], *p* = 0.007). Snakes also demonstrated relatively high repeatability across social contexts (LB compared to MB; Radj|batch = 0.40, 95% CI [0.18, 0.59], *p* < 0.001). Snakes did not show significant repeatability in boldness between the non-social and LB conditions (Radj|batch = 0.02, 95% CI [0, 0.38], *p* = 0.491) but demonstrated marginal repeatability between the non-social and MB conditions (Radj|cond + batch = 0.22, 95% CI [0, 0.49], *p* = 0.078). The low repeatability of behavior across solo and social conditions was the result of a decrease in between-individual variance between the solo and both paired conditions, and an increase in within-individual variance between the solo and LB conditions (Table 1). We additionally tested for repeatability in changes between the non-social trial and the two social trials (ΔSolo-LB vs. ΔSolo-MB). In other words, we tested whether or not individuals that changed a lot in boldness when they were the less bold partner (relative to when tested alone) also changed a lot when they were the more bold partner. Repeatability in change across conditions was high (Radj|batch = 0.50, 95% CI [0.29, 0.57], *p* < 0.001) due to comparable among- and within-individual variance (Table 1).

**Table 1:** Repeatability and variance decomposition for boldness within and across social contexts, and change in boldness across conditions

	Repeatability(*R*_adj_ [95% CI])	Between-individual variance (Mean ± SD)	Within-individual variance (mean ± SD)
Solo	**0.32** [0.07, 0.52]	0.024 ± 0.156	0.051 ± 0.227
LB vs MB	**0.40** [0.29, 0.73]	0.038 ± 0.195	0.057 ± 0.239
Solo vs LB	0.02 [0.00, 0.38]	0.001 ± 0.044	0.077 ± 0.278
Solo vs MB	0.22 [0.00, 0.49]	0.012 ± 0.108	0.042 ± 0.206
ΔSolo-LB vs ΔSolo-MB	**0.50** [0.29, 0.67]	0.069 ± 0.263	0.067 ± 0.259

The first column shows the comparisons (Solo is the non-social condition, LB and MB are the less bold and more bold social conditions). The second column gives the adjusted *R* (*R*_adj_) and 95% confidence intervals (in square brackets). Bolding indicates a significant repeatability value. The third and fourth columns give the mean between- or within-individual variance ± SD

### Plasticity

**Fig. 1:**
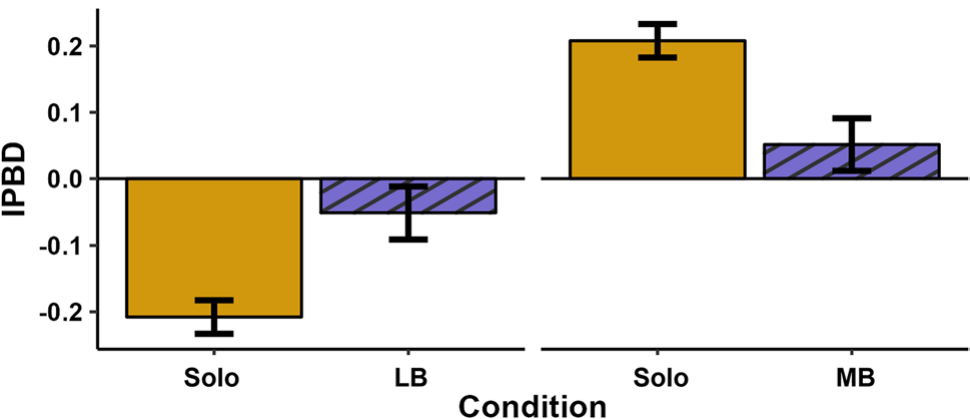
Mean intra-pair boldness difference (IPBD) scores for the solo and paired conditions. Each panel shows the difference in boldness scores between partners when they were tested alone (solid yellow bars) and when they were in a pair (hatched purple bars), either as the less bold (LB, left panel) or more bold (MB, right panel) partner. Error bars are ± SE

As a test of plasticity in boldness, we compared the difference in score between partners in the social conditions (LB and MB) to the expected difference in their scores if they maintained their behavior from the solo trials. We found a significant main effect of social context when comparing the solo and LB conditions (*F*(1,83.95) = 15.82, *p* < 0.001), such that the difference in scores between partners in the LB context was smaller than would be expected based on their solo trials (Fig. 1). In other words, individuals demonstrated plasticity in their boldness, becoming more similar in boldness when together.

**Fig. 2:**
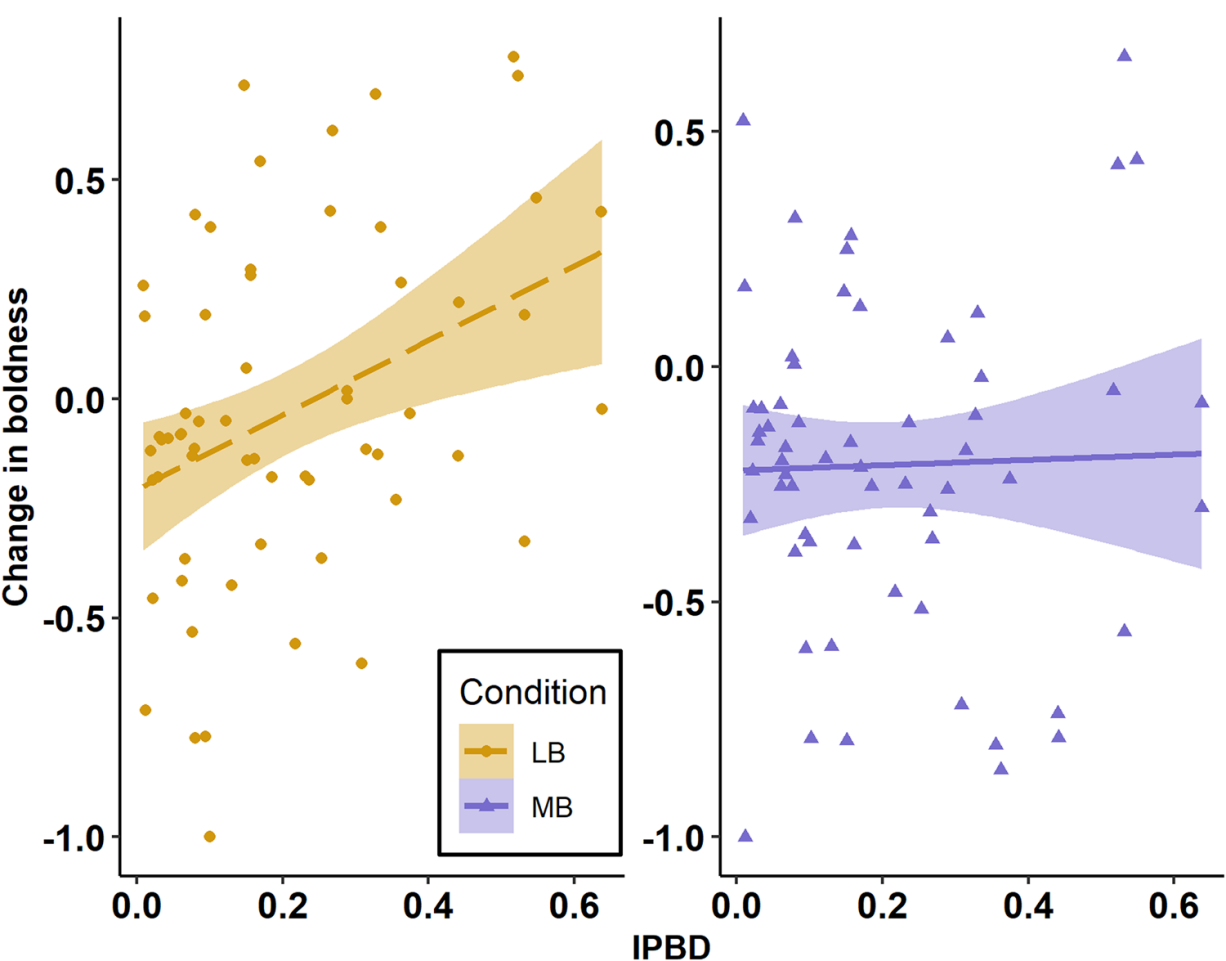
Relationship between change in boldness scores (from solo to paired trials) and intra-pair boldness differences (IPBD), separated into the more bold (MB) and less bold (LB) conditions. Shaded regions show 95% confidence intervals

We found that this plasticity in boldness was a function of the magnitude of the difference in boldness scores within pairs. More specifically, there was a significant effect of the difference in boldness between partners (IPBD) on change in boldness, such that the more an individual's solo boldness score differed from their partner’s score, the more they tended to change during paired testing (*F*(1, 110.38) = 4.37, *p* = 0.039; Fig. 2).

Although the two-way interaction between condition (LB and MB) and difference in boldness (IPBD), when testing for change in boldness, was not significant (*F*(1, 109.69) = 0.350, *p* = 0.555), inspection of the trends across conditions suggested that individuals increased more in boldness when they were the LB partner (*b* = 0.50, 95% CI [− 0.06, 1.07]) than when they were the MB partner (*b* = 0.27, CI [− 0.24, 0.79]). There were no significant main effects of Condition (*F*(1, 84.72) = 1.02, *p* = 0.315), Sex (*F*(1, 50.08) = 1.72, *p* = 0.195), or Weight (*F*(1, 50) = 1.22, *p* = 0.277), and the interaction between Sex and Weight was also not significant (*F*(1, 49) = 2.48, *p* = 0.122). The assumptions of error independences (Durbin–Watson = 2.46) and homogeneity of variance across groups (Levene’s test; Condition *p* = 404; Sex *p* = 513) were both met. Change in boldness did not differ as a function of trial number (*F*(1, 58.74) = 0.56, *p* = 0.457), first partner type (MB or LB; *F*(1, 105.84) = 0.59, *p* = 0.443), nor their interaction (*F*(1, 58.74) = 0.09, *p* = 0.764; Figure S1). This indicates that the plasticity we observed was not being driven by testing order effects. Across pairing types, there was a difference in plasticity. Snakes that were the less bold partner on both trials tended to increase in boldness across both trials, whereas snakes that were the more bold partner twice and mixed-partner snakes (who had one trial MB and one LB), tended to decrease in boldness across trials (main effect of pairing type; *F*(2, 95.06) = 8.49, *p* < 0.001; no pairing type by trial interaction; *F*(2, 58.24) = 0.91, *p* = 0.408; Figure S2). In other words, plasticity was also not the result of alternate pairings, as snakes that were the less bold partner twice tended to increase in boldness twice. Across the MB and LB conditions, we did not find that MB snakes emerged from the shelter significantly sooner (*F*(1, 96.25) = 0.07, *p* = 0.798) or more often (*F*(1, 106.42) = 0.12, *p* = 0.73) than LB snakes (the random effect of testing batch had to be dropped from the latter model due to a singular fit).

## Discussion

We tested eastern gartersnakes for consistency in boldness across social and non-social contexts. We used individual (i.e., solo) boldness test scores to divide the snakes into pairs, such that each snake (where possible) was paired with both a bolder and a less bold partner. In addition, we varied the magnitude of the difference between partners’ boldness scores. We then tested the snakes in their pairs and looked for consistency and plasticity in their boldness scores between the three contexts—solo, social as the less bold partner (LB), and social as the more-bold partner (MB). We hypothesized that, when tested in pairs, snakes would either conform to the boldness level of their partner, or maintain their solo boldness levels. The manner in which the expression of personality traits adapts (or fails to adapt) to changes in social context can help to understand the cognitive processes that apply personality to bias behavior and, hopefully, learn something about how they function and how flexible they are.

We found that boldness was consistent across two solo boldness trials (Table 1), suggesting that our assays capture the effects of a personality trait (see also Skinner and Miller 2022; Skinner et al. 2022). We also found consistent boldness scores across the LB and MB conditions, again suggesting that the behaviors we measured are consistent when snakes are in a social context. However, we found no consistency in boldness across social contexts (between solo and LB or solo and MB). This result suggests that gartersnakes are plastic in their responses to changes in social context, unlike the consistency they display within contexts.

Since we tested most snakes in both the LB and MB conditions, we were also able to test whether snakes show consistency in their plasticity. We found high repeatability in the change in boldness score from solo to social trials (i.e., comparing the change from solo to LB to the change from solo to MB; Table 1). This suggests that plasticity, or behavioral flexibility, is itself a personality trait in our snakes, and that social pressures (e.g., to conform) may have been acting on behavior in conjunction with individual differences in flexibility. Although studied less often than other aspects of personality in animals, individual differences in plasticity are an important component of personality and there is growing evidence that they are widespread across taxa (see Dingemanse et al. 2010 for review). A number of theories have been put forward to explain individual differences in plasticity, including frequency dependent selection (Wolf et al. 2008), and state-dependent plasticity (Wolf and Weissing 2010; Mathot et al. 2011).

The change in boldness that snakes displayed across social contexts acted to decrease the differences between partners, whether the snake was the more or less bold partner. As a result, the difference in boldness between partners was significantly less than would have been expected based on their solo scores (Fig. 1). More specifically, snakes adjusted their behavior to at least partially conform to the boldness level of their partner, particularly when they were the less bold member of the pair (though the trend held for both social conditions). Indeed, we found that the magnitude of plasticity displayed depended on the size of the difference in boldness between the partners: snakes changed their behavior more when they were more different from their partner (Fig. 2).

We conducted solo assessments of boldness first, in arenas containing a single shelter. To reduce the confound of shelter competition during paired testing, we added a second shelter for paired trials. This, along with a general habituation effect, might have been responsible for the decreased boldness that we saw in some snakes in the paired trials. However, this cannot explain the tendency for less bold partners to increase in boldness, and the effects of habituation were mitigated by counterbalancing the order of paired trials. In addition, we found comparable repeatability and between-individual variance within both the solo and paired contexts, which suggests that there was no overall canalization of boldness during the transition from solo to paired trials (Table 1). As research has shown long term boldness consistency in gartersnakes (Brodie 1993; Skinner et al. 2022), we consider it unlikely that any developmental changes contributed to our results. Nevertheless, we cannot completely rule out developmental effects on boldness and hope that future research on snake personality will examine social influences on behavioral plasticity across developmental stages. Our results align with some existing research on conformity, plasticity, and consistency in behavior. Guayasamin et al. (2017), using a similar paradigm to ours, found that less exploratory zebrafish adjusted their behavior to match a more exploratory partner. In other fish species, bold individuals tend to be more consistent across time (*Gasterosteus aculeatus*; Jolles et al. 2019) and social contexts (*Perca fluviatilis*; Magnhagen & Bunnefeld 2009). Similar findings in other taxa suggest that, in general, less bold or exploratory individuals are more likely to adjust their behavior as a function of their social environment (Magnhagen and Bunnefeld 2009; Kurvers et al. 2010; Ólafsdóttir and Magellan 2016), possibly because social facilitation also plays a role in boldness plasticity, with the presence of a conspecific tending to increase boldness generally (e.g., Webster et al. 2007). However, other data imply that both bolder and shyer individuals will conform in a social context (King et al. 2015; Littlewood et al. 2021). Interestingly, we are not aware of any data showing plasticity of behavior used to decrease the similarity of behavior between group members—though there is no a priori reason why this could not occur. It appears that animals are either consistent in their behaviors—often when they are the boldest or most exploratory individual—or change their behaviors to increase conformity within the group. These effects vary by species and likely depend in complex ways on the ecology of the species. For example, individuals may co-conform to the mean when the advantages of expressing a particular personality are superseded by the advantages of group cohesion (Herbert-Read et al. 2013). Given the wide range of habitats and lifestyles inhabited by snakes (Sheehy et al. 2016), a comparison of plasticity in response to social context across snake species would likely help identify the ecological features that help maintain variability in this effect, and the underlying psychological processes that may be conserved.

In many studies on the effects of social context on personality, individuals are often categorized as either bold or shy (Harcourt et al. 2009; King et al. 2015; Frost et al. 2006; Fürtbauer and Fry 2018). Here, by allowing each snake to interact with both bolder and less bold partners, we ensured that the effects we observed result from flexibility in behavior, depending on the direction of the difference between partners. In other words, the same individuals were more flexible when they were the less bold partner and more consistent when they were the bolder partner. This method also reduces the influence of behaviors linked to absolute boldness, as even comparatively shy snakes were still the bolder partner in half of the trials. In addition, this method may be more consistent with natural conditions, as members of fission–fusion or facultative groups would only rarely be the boldest or shyest individual of their group. Uncertainty about the composition of a group could select for behavioral plasticity in systems where competition between individuals is high, and conforming is the best way to ensure access to contested resources (Dingemanse and Wolfe 2013; Dong et al. 2015). For example, gartersnakes cannot share food (Yeager and Burghardt 1991) and compete fiercely for mating opportunities during spring emergence (Friesen et al. 2013; Shine et al. 2001). Flexibility may allow gartersnakes to adjust their boldness to meet the challenges of competition during seasonal social periods (i.e., social competency; Duboscq et al. 2016). During the summer months, when gartersnakes disperse from their den sites and are less social, consistency of personality may help them maintain their social niches and avoid unnecessary competition (a form of social niche specialization; Bergmüller and Taborsky 2010). Some recent data on differential development of personality during the first year of life in male and female gartersnakes—who disperse differently—also supports this conclusion (Skinner et al. 2022). Snakes are often considered cognitively unsophisticated (Turner 1892; Font 2020) and findings like ours, that demonstrate that the cognitive processes underlying the expression of personality traits in snakes are sensitive not only to changes in social context but also to at least some of the specific characteristics of partners, serve to demonstrate that the behaviors of snakes (and other reptiles) are as complex and flexible as they need to be for them to survive in their widely varying environments, and often as sophisticated as those of mammals or birds.

Previous research has shown that gartersnakes respond to the presence of conspecifics (in the laboratory) by aggregating in shelters, and that snakes use a variety of criteria such as shared past experience, sex, relatedness, and diet to choose their aggregation partners (Skinner et al. 2022; Lyman-Henley and Burghardt 1994; Yeager and Burghardt 1991). However, very little is known about how snakes interact when not aggregating under shelters. Here, we demonstrate that snakes adjust their behavior when emerging from a shelter to navigate an open arena so as to conform to their partner, and take into consideration the difference in boldness between them and their partner when doing so. Such situational plasticity has been termed ‘social competency’, as it represents alteration of typical behavior heuristics to the demands of the social context (Duboscq et al. 2016; Taborsky and Oliveira 2012). Social–cognitive perspectives on personality are rare in reptile literature, but offer valuable insight into shared processes across social systems (e.g., Riley et al. 2018). In addition to such situational conformity, individual differences in flexibility influenced behavioral plasticity. Boldness can have a variety of consequences for survival, including altering vulnerability to predation (Magnhagen and Borcherding 2008) and environmental hazards (Bremner-Harrison et al. 2004) or conspecific resource competition (Rudin and Briffa 2011). As a result, plasticity in boldness may be influenced by multiple social and non-social cognitive processes, even in animals such as gartersnakes, that do not form permanent social groups.

## Supplementary Information

Below is the link to the electronic supplementary material.

Supplementary file1 (DOCX 719 KB)

Supplementary file2 (AVI 6307 KB)

Supplementary file3 (AVI 16049 KB)

## Data Availability

All the data reported in this paper are archived at https://osf.io/qv47m/?view_only=8511d9d66b2c4c08b425201368153fad.
