# Supplementary material for: Bolder together: conformity drives behavioral plasticity in eastern gartersnakes
Source: Anim Cogn. 2024 Feb 22;27(1):2. doi: 10.1007/s10071-024-01859-5 (PMC10884060; doi:10.1007/s10071-024-01859-5)

Supplementary Information

**Figure S1**. Video snapshots of the boldness arenas used for the solo (A) and paired (B) trials. The black rectangular objects are shelters. Sheets of plastic were placed over the arena to prevent escapes. Identical styrofoam arenas were used for all trials.


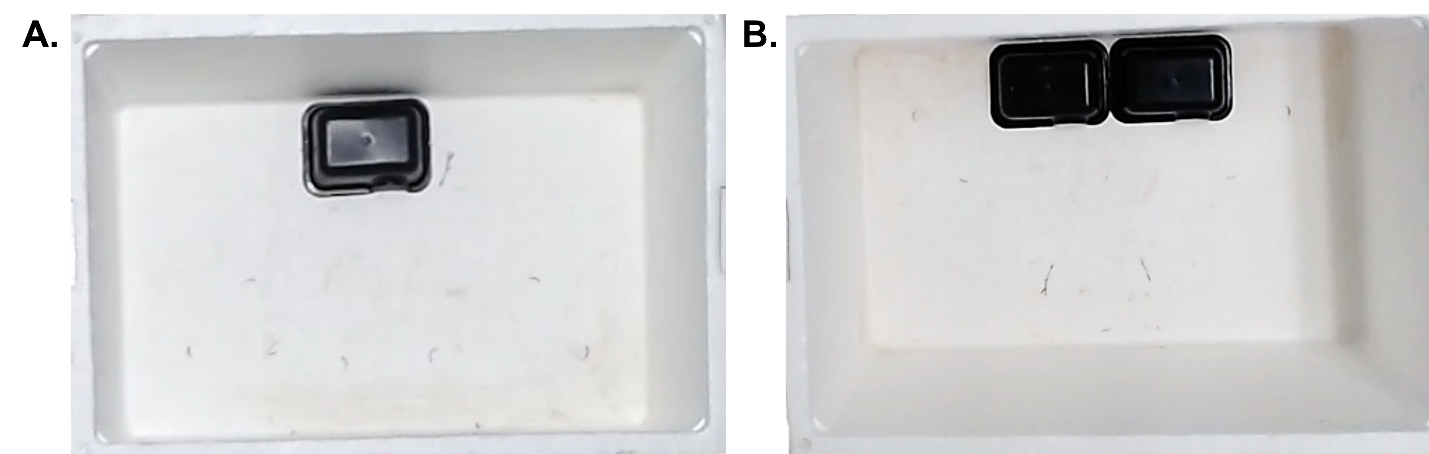


**Video S1**. Sample video of a solo boldness trial. The video starts about 3 minutes after the start of the session, and is sped up by a factor of 4.

**Video S2**. Sample video of a paired boldness trial. The video starts about a minute and a half after the start of the session, and is sped up by a factor of 4.

**Figure S2**. Absolute boldness plasticity across pairing order types and trial numbers. The LB first group were less bold than their first partner whereas the MB First group were bolder than their first partner. Error bars are +/- SE.


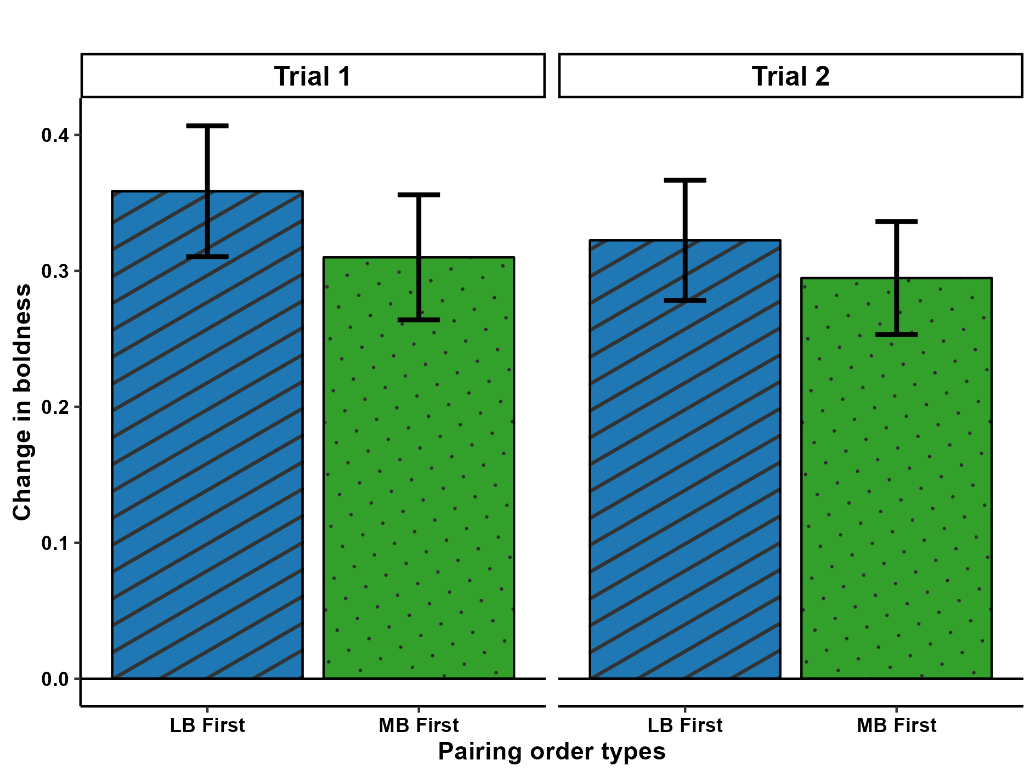


**Figure S2**. Boldness plasticity across pairing types and trial numbers. LB Both snakes were less bold than both of their partners whereas MB Both snakes were the opposite. Snakes in the Mixed group were less bold than one partner and bolder than the other.

Error bars are +/- SE.


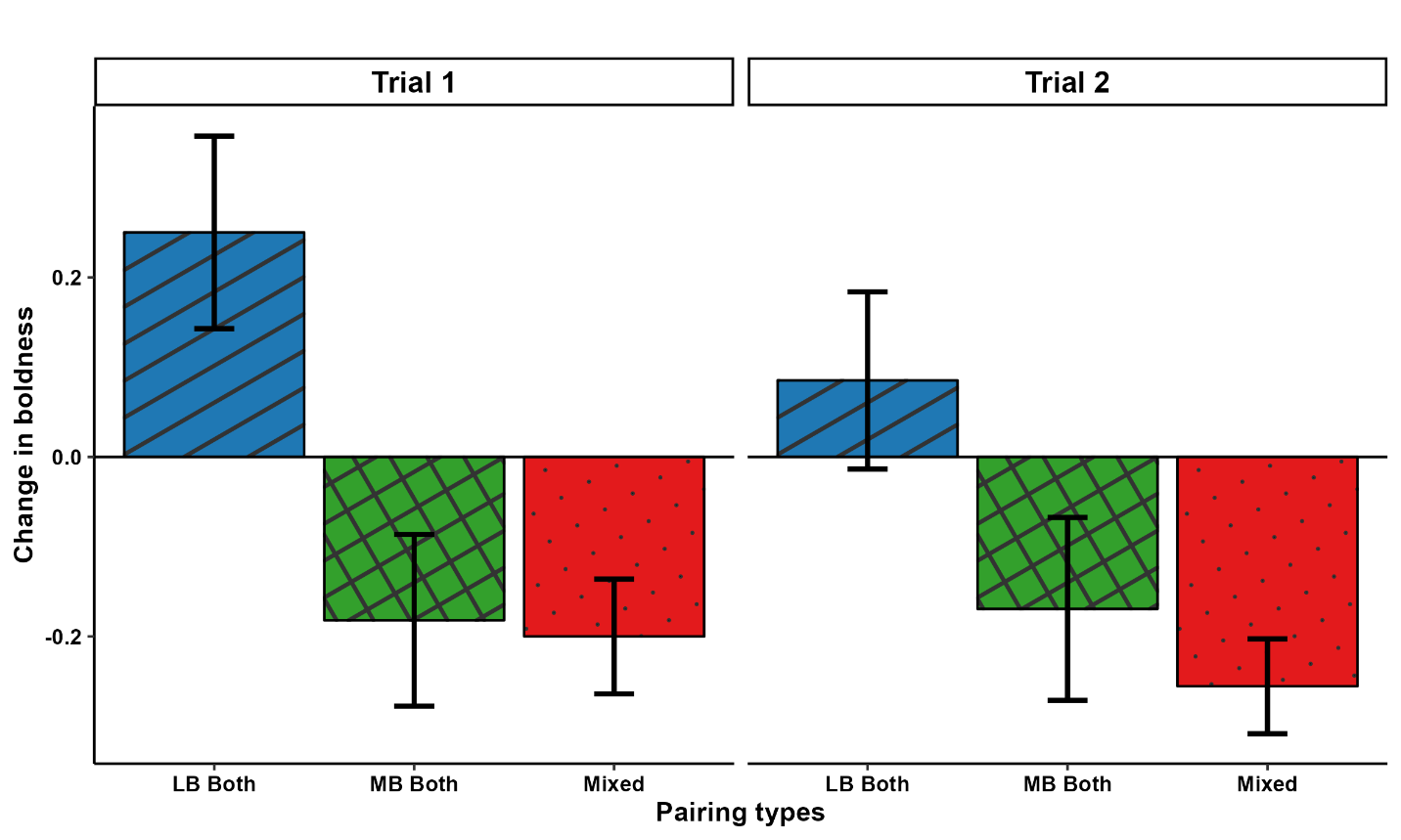

Supplement: Supplementary file 1 — Supplementary file1 (DOCX 719 KB) [file 10071_2024_1859_MOESM1_ESM.docx]
